# Effect of a Single Immersive Virtual Nature Session on Preoperative Surgical Fear and State Anxiety in Patients Awaiting a Laparoscopic Sleeve Gastrectomy: A Randomised Controlled Trial

**DOI:** 10.3390/healthcare14183067

**Published:** 2026-09-17

**Authors:** Dilek Güneş, Özlem Acar

**Affiliations:** 1 Department of Surgical Nursing, Faculty of Health Sciences, Fırat University, 23119 Elazığ, Türkiye; 2 Provincial Health Directorate, 23050 Elazığ, Türkiye; ozlemaccar@gmail.com

**Keywords:** bariatric surgery, sleeve gastrectomy, preoperative anxiety, surgical fear, virtual reality, perioperative nursing

## Abstract

**Background:** Patients awaiting bariatric surgery face permanent anatomical change, lifelong behavioural adaptation, and uncertainty about weight-loss maintenance; consequently, they report high levels of surgical fear and state anxiety. Immersive 360° virtual nature exposure delivered through a head-mounted display is a low-cost, nurse-deliverable, non-pharmacological option, but its effect on surgical fear specifically, and in bariatric candidates in particular, is largely untested. The aim was to determine whether a single 20 min session delivered in the immediate preoperative period reduces surgical fear and state anxiety in patients scheduled for laparoscopic sleeve gastrectomy. **Methods:** A single-centre, two-arm, parallel-group randomised controlled trial was conducted in a general surgery clinic between March 2025 and June 2026 and is reported in accordance with the CONSORT 2025 statement (ClinicalTrials.gov NCT07774156; registered retrospectively on 18 August 2026). One hundred and twenty adults scheduled for laparoscopic sleeve gastrectomy were randomly allocated 1:1, using sequentially numbered opaque sealed envelopes, to the intervention or to routine care. Two hours before transfer to theatre, the intervention arm viewed a single 20 min 360° nature video with a natural soundtrack through a head-mounted display; the control arm received the standard clinical protocol. Premedication was administered after post-testing in both arms. Surgical fear (Surgical Fear Questionnaire, SFQ) and state anxiety (State Anxiety Inventory, STAI-S) were measured before and after the intervention period. The primary analysis was a baseline-adjusted comparison of post-test SFQ; because the homogeneity-of-regression-slopes assumption was violated, a group-by-baseline interaction model was used, and conditional treatment effects were reported. All other analyses were exploratory. **Results:** All 120 patients completed the trial. In the interaction model, the estimated control minus intervention difference in post-test SFQ was significant across the entire observed baseline range, from 11.43 points (95% CI 5.59 to 17.27) at a baseline score of 0 to 26.37 points (95% CI 18.35 to 34.40) at a baseline score of 80, and was 17.19 points (95% CI 13.38 to 20.99) at the sample mean baseline. Post-test state anxiety was lower in the intervention arm by 24.87 points after baseline adjustment (95% CI −27.33 to −22.41; F(1, 117) = 400.84, *p* < 0.001). Surgical fear fell by 8.68 points in the intervention arm and rose by 8.22 points in the control arm; state anxiety fell by 18.75 points and rose by 7.15 points, respectively. Forty-five per cent of participants gave identical responses to all eight SFQ items at post-test, and when these participants were excluded, the between-group difference in post-test SFQ fell to 10.66 points (*p* = 0.029), and the baseline interaction disappeared. **Conclusions:** A single 20 min session of immersive 360° virtual nature exposure was followed by substantially lower surgical fear and state anxiety than routine care, under which both increased over the same interval. The magnitude of these effects greatly exceeds pooled estimates from the existing literature and should not be attributed to the virtual reality content itself: the comparator was routine care rather than an attention-matched control, so the contrast reflects a bundle of immersive stimulation, novelty, protected quiet time, and individual researcher attention; the trial was unblinded with self-reported outcomes; and a substantial response-set pattern in the SFQ data may have inflated the apparent effect on surgical fear. The findings support further evaluation in attention-controlled trials rather than immediate adoption.

## 1. Introduction

Obesity has become one of the defining public health problems of the present century. Global adult obesity prevalence rose from 6.4% in 1990 to 16.1% in 2022 and is projected to reach almost 39% by 2040 [1], and the number of adults living with obesity is expected to exceed 1.1 billion by 2030 [2]. The condition is causally linked to cardiovascular disease, type 2 diabetes, several cancers, and premature mortality, and its management now consumes a substantial share of health system resources worldwide [3].

Among available treatments, metabolic and bariatric surgery produces the largest and most durable weight loss and the most consistent improvement in obesity-related comorbidities, and it is recommended for adults with a body mass index of 35 kg/m^2^ or above and, in selected cases, from 30 kg/m^2^ [4,5]. Yet bariatric surgery is not simply a metabolic procedure. It permanently alters gastrointestinal anatomy and physiology [6], imposes lifelong dietary restriction and behavioural adaptation, and confronts patients with rapid changes in body image and with uncertainty about whether weight loss can be sustained [7]. These features make the preoperative period a psychologically demanding one, distinct in character from that preceding other elective abdominal operations.

The psychological burden of the preoperative period has measurable clinical consequences. A recent systematic review and meta-analysis found preoperative anxiety to be associated with prolonged recovery from anaesthesia, delayed extubation and greater propofol consumption [8], and a further meta-analysis identified it as a risk factor for chronic post-surgical pain [9]. Alongside general state anxiety, a more specific construct has been described: surgical fear, defined as the patient’s apprehension about the operation itself and about its short- and long-term consequences, and measurable with the Surgical Fear Questionnaire [10]. In bariatric populations, preoperative surgical fear has been shown to correlate with postoperative pain intensity and impaired sleep quality [11], and both fear and anxiety are higher in patients who lack information about the procedure they are about to undergo [12]. Reducing fear and anxiety before bariatric surgery is therefore not only a matter of comfort but a plausible route to better perioperative outcomes.

Pharmacological anxiolysis remains the most common response, but its costs are increasingly recognised: perioperative benzodiazepine administration has been associated with poorer patient-reported recovery outcomes in a meta-analysis of randomised trials [13]. Non-pharmacological interventions, which nurses can deliver independently and whose adverse-effect profile is generally mild, although discomfort and cybersickness are recognised possibilities, have therefore attracted growing attention as first-line alternatives [14,15].

Immersive virtual reality, and in particular 360° virtual nature exposure delivered through a head-mounted display, is among the most promising of these. By occupying the visual and auditory channels with an absorbing alternative environment, VR limits the attentional resources available for threat appraisal and rumination. The rationale is grounded in Attention Restoration Theory, which holds that environments that are engaging yet undemanding, of which natural scenery is the archetype, permit recovery of directed attention and a reduction in negative affect [16]; electrophysiological work has since shown involuntary attention restoration during exposure to mobile-based 360° virtual nature [17], and experimental studies indicate that immersive 360° nature can reproduce much of the mood benefit of outdoor exposure [18]. Clinically, meta-analyses report moderate to large reductions in preoperative anxiety with VR-enhanced interventions [14], although effects in adults have been less consistent than in children [19], and individual randomised trials in elective surgery [20], colorectal and abdominal wall surgery [21], open-heart surgery [22], cardiac surgery [23] and gynaecological oncology [24] have generally, though not universally, favoured VR.

Two gaps remain. First, most trials have measured general state anxiety alone; surgical fear, which is more specific to the operation and its consequences and which behaves differently from general anxiety, has rarely been an outcome, and the few trials that have measured it have not examined its short- and long-term components separately. Second, bariatric surgery candidates are almost absent from this literature. The one randomised trial of VR in this population applied the intervention after laparoscopic sleeve gastrectomy, targeting postoperative pain and anxiety in intensive care [25]; the preoperative period, when fear and anxiety are at their peak and when a brief nursing intervention would be most feasible, has not been studied. The present trial was designed to address both gaps.

This trial aimed to determine the effect of a single preoperative immersive VR session on surgical fear and state anxiety in patients scheduled for bariatric surgery. Two hypotheses were tested: (H1) VR reduces surgical fear relative to routine care; and (H2) VR reduces state anxiety relative to routine care.

## 2. Materials and Methods

### 2.1. Study Design, Setting and Trial Registration

**Figure 1:**
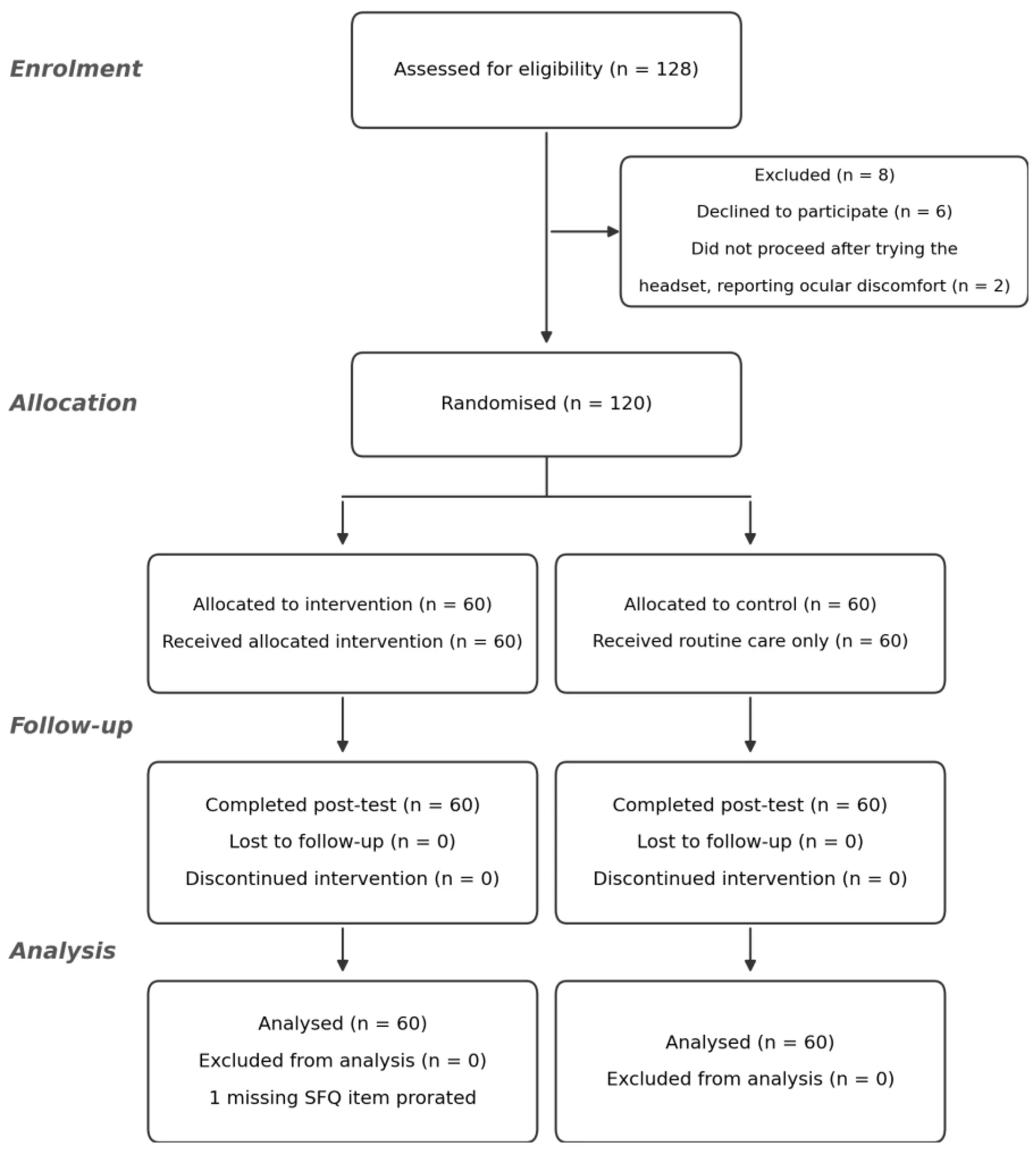
CONSORT flow diagram of participant recruitment, allocation, follow-up and analysis.

This was a single-centre, two-arm, parallel-group randomised controlled trial with a pre-test/post-test design and 1:1 allocation, conducted in the General Surgery Clinic of a hospital in eastern Türkiye between March 2025 and June 2026. The trial is reported in accordance with the CONSORT 2025 statement for reporting randomised trials [26]. The participant flow is shown in Figure 1. The trial was registered at ClinicalTrials.gov (NCT07774156) on 18 August 2026. Registration was retrospective: it took place after recruitment and outcome data collection had been completed and was undertaken at the point of journal submission in order to meet the journal’s requirements. The delay was not an oversight in the conduct of the study but reflects the regulatory context in which it was carried out. Prospective registration in a public clinical trials registry is not a condition of ethical approval or of institutional authorisation for nursing intervention research in Türkiye, and the study was designed and approved without it. The trial was nevertheless designed as a randomised controlled trial from the outset: the two-arm parallel design, the allocation of 60 participants to each arm, the intervention and the primary outcome were specified in the protocol reviewed and approved by the Non-Interventional Research Ethics Committee of Fırat University on 30 January 2025, approximately two months before the first participant was enrolled. Because the registry entry itself post-dates data collection, it cannot serve as evidence that the particular statistical models and sensitivity analyses reported here were specified in advance, and Section 2.10 therefore distinguishes the primary analysis from analyses that were exploratory or prompted by observed characteristics of the data. No separate written statistical analysis plan was prepared beyond the outcome and comparison specified in the protocol.

### 2.2. Participants

The target population comprised all adults scheduled for elective laparoscopic sleeve gastrectomy in the study clinic during the recruitment period; all 120 participants underwent this single procedure, so the sample is procedurally homogeneous. Eligible patients were aged 18 years or older, with no upper age limit applied, able to communicate verbally, without visual or hearing impairment, without a diagnosed psychiatric disorder, and not concurrently using any other non-pharmacological anxiety-reduction method. Psychiatric status was established by review of the hospital medical record rather than by structured diagnostic interview or a screening instrument, and patients with a documented psychiatric diagnosis were not approached; information on psychotropic medication was not extracted separately. Because psychiatric comorbidity is common among bariatric candidates, this exclusion restricts generalisability to a psychiatrically unselected bariatric population, and this is acknowledged in the Limitations. Patients younger than 18 years, those with visual or hearing impairment, and those using another non-pharmacological method during the study period were excluded. All participants gave written and verbal informed consent before randomisation.

### 2.3. Sample Size

The sample size was calculated a priori with G*Power 3.1.9.7 (Heinrich Heine University Düsseldorf, Düsseldorf, Germany) [27]. An effect size of d = 0.70 was assumed, taken from the randomised trial of virtual reality in patients undergoing laparoscopic sleeve gastrectomy that was available when the study was planned [25]. Two features of this choice should be stated plainly. First, the significant between-group difference in that trial was obtained for postoperative pain rather than for anxiety, for which no significant difference was found; the assumed effect size therefore derives from a different outcome, measured after surgery rather than before it. Second, the present primary outcome is preoperative surgical fear, a construct not assessed in that trial. The assumption is nevertheless close to the pooled estimate subsequently reported for virtual reality and preoperative anxiety in adults (g ≈ 0.76) [14], which supports its magnitude even though its source was imperfect. With α set at 0.05 (two-sided) and power at 95%, a minimum of 54 participants per arm was required; 60 were recruited into each arm to allow for attrition, giving 120 in total.

### 2.4. Randomisation, Allocation Concealment and Blinding

The allocation sequence was generated before recruitment began using the Research Randomizer web application (Social Psychology Network, Middletown, CT, USA), which partitioned the integers 1 to 120 into two sets of 60. A single coin toss, performed before any patient was enrolled and before any allocation was revealed, determined that the first set would constitute the intervention arm; because this toss preceded all enrolment, it has no bearing on the validity of the randomisation. The sequence was generated by the first author (D.G.), who also prepared the envelopes and retained the allocation list, which was not accessible to the researcher who recruited patients. Allocations were concealed in sequentially numbered, opaque, sealed envelopes, which were opened in order only after a patient had been assessed as eligible and had given written consent. The person recruiting patients therefore had no foreknowledge of the next assignment, and eligibility decisions could not have been influenced by upcoming allocations. Recruitment, eligibility assessment, opening of the allocation envelope, delivery of the intervention and collection of outcome data were all carried out by the second author (Ö.A.). Because the intervention was an overt behavioural procedure and both outcomes were patient-reported, neither participants nor the researcher delivering the intervention could be blinded; the trial was therefore open-label. Allocation was concealed at the point of enrolment, but outcome assessment was not independent of intervention delivery, since the same researcher performed both. The implications are considered in Section 2.9 and Section 4.5.

### 2.5. Outcomes

The primary outcome was surgical fear, measured as the SFQ total score at post-test. The secondary outcome was state anxiety measured with the STAI-S. The two SFQ subscale scores, covering fear of the short-term and of the long-term consequences of surgery, were examined as exploratory outcomes. All outcomes were assessed immediately before the intervention period (pre-test) and after it (post-test).

### 2.6. Measurement Instruments

Personal Information Form: A form developed by the researchers from the relevant literature, recording sociodemographic characteristics (age, sex, marital status, educational level, perceived income), anthropometric data (height, weight, body mass index), and obesity-related and clinical characteristics (comorbid disease, duration of overweight, duration of weight-loss attempts, weight-loss methods used, previous surgery, prior knowledge and prior use of virtual reality, and the source of information about the planned operation).

Surgical Fear Questionnaire (SFQ): Developed by Theunissen et al. to quantify fear in patients awaiting elective surgery [10] and adapted into Turkish by Bağdigen and Karaman Özlü [28]. Eight items are rated on an 11-point numeric rating scale anchored at 0 (“not afraid at all”) and 10 (“very afraid”). Items 1–4 form the short-term fear subscale and items 5–8 the long-term fear subscale; each subscale ranges from 0 to 40 and the total from 0 to 80. Cronbach’s α was 0.93 in the original validation study [10]. In the present sample, it was 0.98 at pre-test and 0.99 at post-test; the interpretation of these unusually high values is addressed in Section 3.7 and Section 4.5.

State Anxiety Inventory (STAI-S): Developed by Spielberger et al. [29] and adapted into Turkish by Öner and Le Compte [30]. Twenty items are rated from 1 to 4, of which 10 are reverse-worded. The total ranges from 20 to 80, with higher scores indicating greater state anxiety. Cronbach’s α was 0.83 in the adaptation study [30]. In the present sample, it was 0.90 at pre-test and 0.97 at post-test.

### 2.7. Intervention

Patients allocated to the intervention arm received a single session two hours before transfer to the operating theatre. After the pre-test measures had been completed, the headset was introduced and demonstrated. A VR Shinecon G04ea head-mounted display (Dongguan Shinecon Digital Device Co., Ltd., Dongguan, China) with integrated headphones, fitted with the researcher’s mobile telephone, was used. All participants viewed the same 360° nature video, comprising forest, sea, and landscape scenes with an accompanying natural soundtrack; content was not selected by the patient or the researcher and did not vary between participants. The stimulus was a publicly available YouTube video entitled “Son Çalışma” (video identifier cMK08aolmD8; https://youtu.be/cMK08aolmD8 (accessed on 1 March 2025)), with a total running time of 30 min and 35 s, of which the first 20 min were shown; playback was stopped at 20 min in every case. The video was played through the YouTube application on an iPhone 14 Pro Max (Apple Inc., Cupertino, CA, USA) mounted in the head-mounted display. The video was available throughout the data collection period but has since been removed by its uploader and is no longer retrievable; the identifier, running time and content description are given so that the stimulus can be documented and an equivalent selected by others. The material was monoscopic 360° video rather than an interactive virtual environment: head movement altered the viewpoint within the scene, but participants could not otherwise interact with the content. Audio was delivered through the headphones integrated into the device. Participants were seated or semi-recumbent in their own room, from which noise and interruptions were excluded, and the researcher remained present throughout to monitor the participant and assist with the device. For these reasons, the intervention is described more precisely as immersive 360° virtual nature exposure delivered through a head-mounted display than as interactive virtual reality. A duration of 20 min was chosen because relaxation-focused sessions of roughly 15 to 40 min are those most commonly reported to be effective and well tolerated, whereas longer exposures increase the risk of cybersickness [31]. Post-test measures were administered 10 min after the session ended. Adverse effects were not elicited using a predefined checklist or a structured tolerability questionnaire; they were recorded only when reported spontaneously by the patient or observed by the researcher. No adverse effect was reported or observed among the 60 patients who received the intervention, and no session was interrupted. Because ascertainment was passive, this should not be read as evidence that the intervention is free of adverse effects; two otherwise eligible patients reported ocular discomfort when the device was demonstrated before enrolment and did not take part (Figure 1).

### 2.8. Control Condition and Co-Interventions

Patients allocated to the control arm received the standard preoperative protocol of the unit, which comprised routine nursing preparation for surgery, standard preoperative information delivered by the surgical and nursing team, and routine physiological monitoring. No additional structured contact, information-giving, or attention was provided during the interval between the two assessments, and no attention-matched activity was substituted for the intervention session. Pre-test measures were administered two hours before transfer to theatre and post-test measures approximately 30 min later, so that the interval between assessments matched that in the intervention arm. Premedication was administered to all patients in both arms according to the unit’s standard anaesthetic protocol, and in every case after the post-test measures had been completed. No anxiolytic, sedative, analgesic or other psychoactive medication was therefore in effect at either assessment point in either arm, and pharmacological co-intervention cannot account for the observed between-group difference. The comparator was routine care and not an attention-matched or sham control.

### 2.9. Data Collection Procedure

All data were collected by the second author (Ö.A.) through face-to-face interview. Both instruments were interviewer-administered: the researcher read each item aloud to the participant and recorded the response on the form, and was therefore able to see each response as it was given. Questionnaires were not self-completed. The same procedure was used identically in both arms. The Personal Information Form, the SFQ, and the STAI-S were administered at pre-test, and the SFQ and STAI-S were re-administered at post-test. Each form was checked for completeness in the presence of the participant before the encounter ended, which accounts for the very low rate of missing data. Because the same unblinded researcher delivered the intervention and administered the outcome measures, and because administration was by interview rather than self-report, outcome ascertainment was not independent of intervention delivery; the implications are considered in Section 4.5.

### 2.10. Statistical Analysis

Data were analysed with SPSS version 25.0 (IBM Corp., Armonk, NY, USA). Analyses followed the intention-to-treat principle; no participant was lost to follow-up, and all 120 randomised patients were analysed in the arm to which they were allocated. Across 6720 item responses (120 participants, two instruments, two time points), a single item was missing (0.015%), on the pre-test SFQ of one participant. Questionnaires were completed in the presence of the researcher, who checked each form for completeness before the participant left, which accounts for the very low rate of missing data. The missing item was handled by person-mean prorating, in which the mean of the seven observed SFQ items was multiplied by eight; a complete-case sensitivity analysis gave materially identical results.

Because the trial was registered only after data collection had been completed, no analysis can be described as prospectively specified on the basis of the registry entry. The following hierarchy is therefore stated explicitly. The primary analysis was the baseline-adjusted between-group comparison of post-test SFQ total score, the primary outcome named in the ethics-approved protocol. The corresponding analysis of post-test STAI-S was the single secondary analysis. We do not describe either as confirmatory in the prespecification sense, because no analysis plan predating inspection of the data exists; the protocol establishes the outcome and the comparison, not the particular statistical models used to estimate them. All remaining analyses reported below, namely the SFQ subscale analyses, the mixed analysis of variance, change-score comparisons, within-group tests, correlations, the Reliable Change Index classifications, the moderation analysis, and every sensitivity analysis, are exploratory or supportive and are reported as such. No correction for multiplicity was applied because only one primary comparison and one secondary comparison were made; the *p*-values attached to exploratory analyses are descriptive and should not be read as independent confirmations of efficacy.

Continuous variables are summarised as means and standard deviations and categorical variables as frequencies and percentages. Internal consistency in the present sample was quantified with Cronbach’s alpha. Normality was examined with the Shapiro–Wilk test together with skewness and kurtosis coefficients, and homogeneity of variance with Levene’s test. Baseline sociodemographic and clinical characteristics were summarised descriptively by randomised group; no null-hypothesis significance tests were used to assess baseline comparability. Observed imbalances judged potentially relevant to outcome interpretation were examined in sensitivity analyses.

The primary analysis was an analysis of covariance of the post-test score with group as a fixed factor and the corresponding pre-test score as a covariate, the recommended approach for two-arm pre-test/post-test trials [32]. Homogeneity of regression slopes was tested by adding the group-by-covariate interaction term. For surgical fear, this assumption was violated, and the interaction model was therefore adopted as the primary model for that outcome. Because a single constant coefficient cannot represent a treatment effect that varies with baseline, the full interaction model is reported with coefficients, standard errors, and confidence intervals, and conditional treatment effects with 95% confidence intervals are given at interpretable baseline values across the observed range, together with a Johnson–Neyman analysis of the region of significance. Group was coded 0 for the intervention arm and 1 for the control arm, and the covariate was mean-centred, so that a positive group coefficient indicates a higher (worse) post-test score under routine care. The multivariable model for surgical fear retains the same interaction term.

Supportive analyses comprised independent-samples *t* tests at each time point and on change scores (with Welch’s correction where Levene’s test indicated unequal variances), paired-samples* t* tests within groups, a 2 × 2 mixed analysis of variance testing the time by group interaction, Mann–Whitney U and Wilcoxon signed-rank repetitions of every parametric comparison, a rank-transformed analysis of covariance, non-parametric bootstrap resampling with 10,000 replications, and multivariable linear regression adjusted for age, sex, body mass index and previous surgery, with multicollinearity assessed by variance inflation factors and tolerance and residual independence by the Durbin–Watson statistic. Associations between surgical fear and state anxiety were quantified with Pearson’s correlation coefficient, with Fisher z-transformed confidence intervals, and with Spearman’s rank correlation as a sensitivity analysis.

Inspection of the item-level data showed that a substantial proportion of participants had given identical responses to all eight SFQ items. The analyses that follow from this observation were therefore post hoc and were prompted by the data rather than planned. The proportion of uniform responders was quantified at each time point and separately for uniform zero and uniform non-zero patterns, and the primary baseline-adjusted analysis was repeated in the corresponding subsamples so that the same analytical framework is applied throughout.

Effect sizes are reported as Hedges’ g with 95% confidence intervals for between-group comparisons, Cohen’s dz for within-group change, and partial eta squared for analysis-of-variance terms, using the conventional benchmarks [33] only as a frame of reference. Given the design limitations set out in Section 4.5, confidence intervals rather than conventional qualitative descriptors should carry the interpretation of these effect sizes. The Reliable Change Index was computed for both instruments [34] as an exploratory analysis. Cronbach’s alpha quantifies internal consistency and not the stability of repeated measurement, and using it in the Reliable Change Index formula understates measurement error and makes the threshold for reliable improvement permissive. The index was therefore computed twice, once using Cronbach’s alpha and once using the pre-test to post-test correlation within the control arm. Neither coefficient is a satisfactory estimate of test–retest reliability, because the control arm did not remain stable over the interval, and no independently established test–retest coefficient for the SFQ in a comparable population was available. The individual-level classification for surgical fear is therefore reported as Table S1 rather than in the main text, and only the state anxiety classification, which is far less sensitive to the coefficient used, is retained here. All tests were two-sided, and significance was set at *p* < 0.05.

### 2.11. Ethical Considerations

Ethical approval was granted by the Non-Interventional Research Ethics Committee of Fırat University on 30 January 2025, approximately two months before the first participant was enrolled (decision number 2025/02-53), and institutional permission was obtained from the participating hospital. The committee decision is available from the corresponding author on request. Regarding the remit of that committee, in Türkiye the designation “interventional” in ethics committee nomenclature refers specifically to research on medicinal products, biological products and medical devices, which is reviewed by designated clinical research ethics committees. Trials of behavioural, educational and nursing interventions that involve none of these products fall within the remit of non-interventional research ethics committees, which are the competent bodies for studies of this type. The committee reviewed the full protocol, including the randomised allocation of patients to the intervention or to routine care, and the head-mounted display used was a consumer entertainment device rather than a medical device. All patients gave written and verbal informed consent after being informed of the purpose and procedures of the study and of their right to withdraw at any time without consequence for their clinical care. The study was conducted in accordance with the principles of the Declaration of Helsinki.

## 3. Results

### 3.1. Participant Flow and Baseline Characteristics

Of 128 patients who met the eligibility criteria, six declined to take part, and two did not proceed after the head-mounted display was demonstrated, reporting ocular discomfort. The remaining 120 patients were randomised, 60 to the intervention arm and 60 to the control arm. No participant subsequently withdrew or was lost to follow-up, and all 120 completed both assessments (Figure 1).

All participants were scheduled for laparoscopic sleeve gastrectomy. The mean age of the sample was 35.68 ± 9.85 years, and the observed age range was 20 to 59 years; no upper age limit was applied as an eligibility criterion. Mean body mass index was and the mean body mass index was 44.34 ± 4.26 kg/m^2^; 86 patients (71.7%) were women and 108 (90.0%) were classified as morbidly obese. Baseline characteristics are presented descriptively in Table 1. Age, body mass index, body weight, and height were similar across the randomised groups. The most notable observed imbalances were in previous surgery (38.3% in the intervention arm vs 15.0% in the control arm) and in the distribution of weight-loss methods; these variables were therefore included in additional sensitivity analyses without using baseline significance testing as a selection criterion.

### 3.2. Surgical Fear and State Anxiety Before and After the Intervention

At pre-test, mean surgical fear was 29.88 ± 26.68 in the intervention arm and 31.75 ± 26.70 in the control arm; mean state anxiety was 50.83 ± 6.98 and 47.03 ± 7.68, respectively. The observed difference in baseline state anxiety was addressed in the baseline-adjusted analyses.

**Figure 2:**
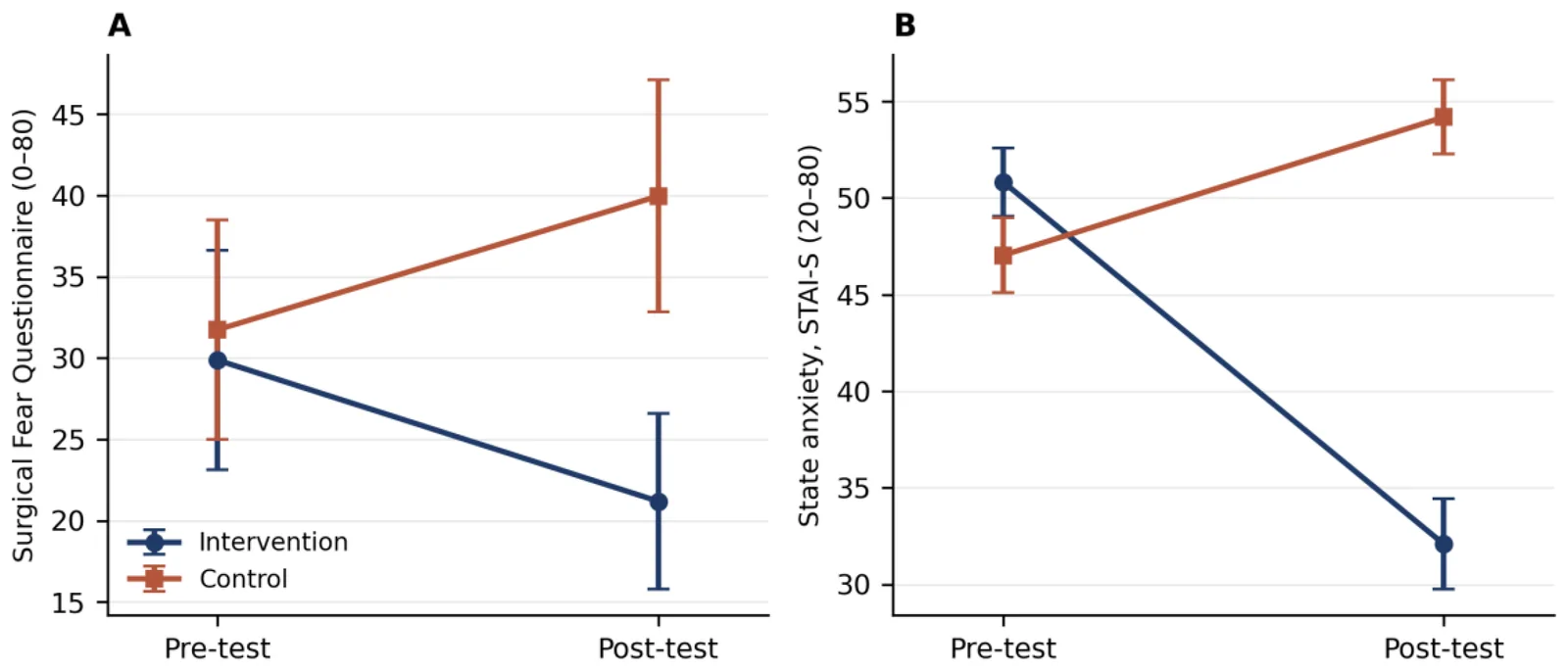
Mean Surgical Fear Questionnaire (**A**) and State Anxiety Inventory (**B**) scores at pre-test and post-test by group. Error bars represent 95% confidence intervals.

At post-test, the pattern was reversed, and the differences were large. Surgical fear was lower in the intervention arm (21.20 ± 21.44 vs. 39.97 ± 28.20; Welch t(110.1) = −4.104, *p* < 0.001, g = −0.74, 95% CI −1.11 to −0.37), as was state anxiety (32.08 ± 9.27 vs. 54.18 ± 7.58; t(118) = −14.300, *p* < 0.001, g = −2.59, 95% CI −3.08 to −2.11). These very large standardised effects should be interpreted through their confidence intervals and in the light of the design limitations set out in Section 4.5 rather than through conventional descriptive labels. Results are given in Table 2 and illustrated in Figure 2.

**Figure 3:**
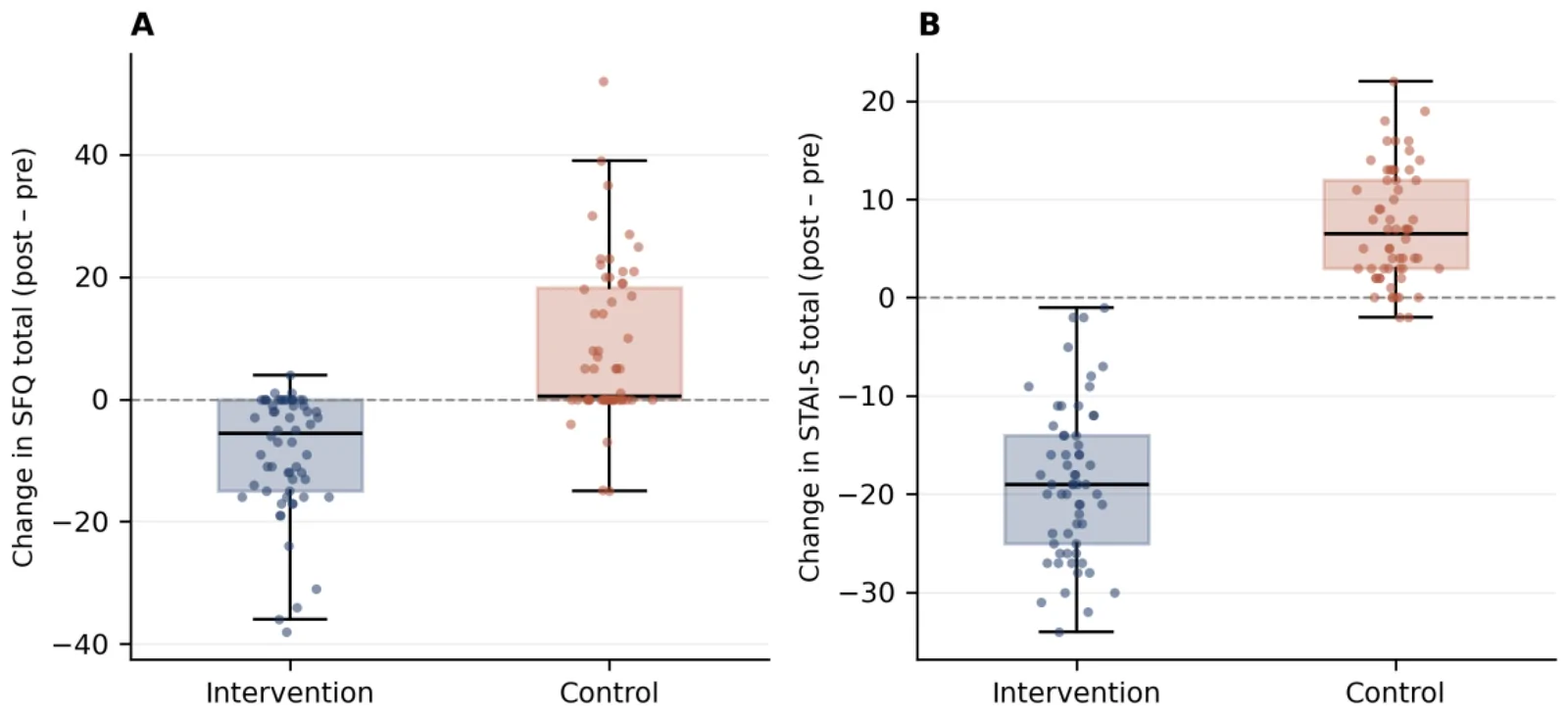
Distribution of individual change scores (post-test minus pre-test) for surgical fear (**A**) and state anxiety (**B**) by group. Boxes show the median and interquartile range; individual patients are plotted as points. The dashed line marks no change.

The two arms moved in opposite directions. In the intervention arm, surgical fear fell by 8.68 points (95% CI −11.23 to −6.14) and state anxiety by 18.75 points (95% CI −20.75 to −16.75). In the control arm, surgical fear rose by 8.22 points (95% CI 4.89 to 11.54) and state anxiety by 7.15 points (95% CI 5.65 to 8.65). The between-group difference in change was −16.90 points (95% CI −21.00 to −12.80) for surgical fear and −25.90 points (95% CI −28.35 to −23.45) for state anxiety (Figure 3). Within-group tests are reported for completeness in Table 2 but are not informative about treatment efficacy, which rests on the randomised between-group contrast.

### 3.3. Baseline-Adjusted Analysis

For surgical fear, the assumption of homogeneous regression slopes was violated (group by covariate interaction *p* = 0.011), and the interaction model was therefore adopted as the primary model for this outcome. Its coefficients are given in Table 3. With group coded 0 for the intervention arm and 1 for the control arm and the covariate mean-centred at a baseline SFQ score of 30.82, the group coefficient was 17.19 (SE 1.92, 95% CI 13.38 to 20.99, *p* < 0.001) and the interaction coefficient was 0.187 (SE 0.073, 95% CI 0.043 to 0.330, *p* = 0.011). The model explained 84.4% of the variance in post-test scores (adjusted R^2^ = 0.844).

**Table 3:** Primary interaction model for post-test surgical fear and conditional treatment effects across the observed baseline range.

Model Term or Baseline Value	B or Difference	SE	95% CI	*p*
Interaction model (adjusted R^2^ = 0.844)				
Intercept (intervention arm, at mean baseline)	21.90	1.36	19.21 to 24.59	<0.001
Group (control vs. intervention)	17.19	1.92	13.38 to 20.99	<0.001
Baseline SFQ (mean-centred)	0.755	0.051	0.653 to 0.856	<0.001
Group × baseline SFQ	0.187	0.073	0.043 to 0.330	0.011
Conditional control minus intervention difference				
at baseline SFQ = 0	11.43	2.95	5.59 to 17.27	<0.001
at baseline SFQ = 20	15.17	2.08	11.06 to 19.28	<0.001
at baseline SFQ = 30.82 (sample mean)	17.19	1.92	13.38 to 20.99	<0.001
at baseline SFQ = 50	20.77	2.37	16.07 to 25.47	<0.001
at baseline SFQ = 80	26.37	4.05	18.35 to 34.40	<0.001

**Figure 4:**
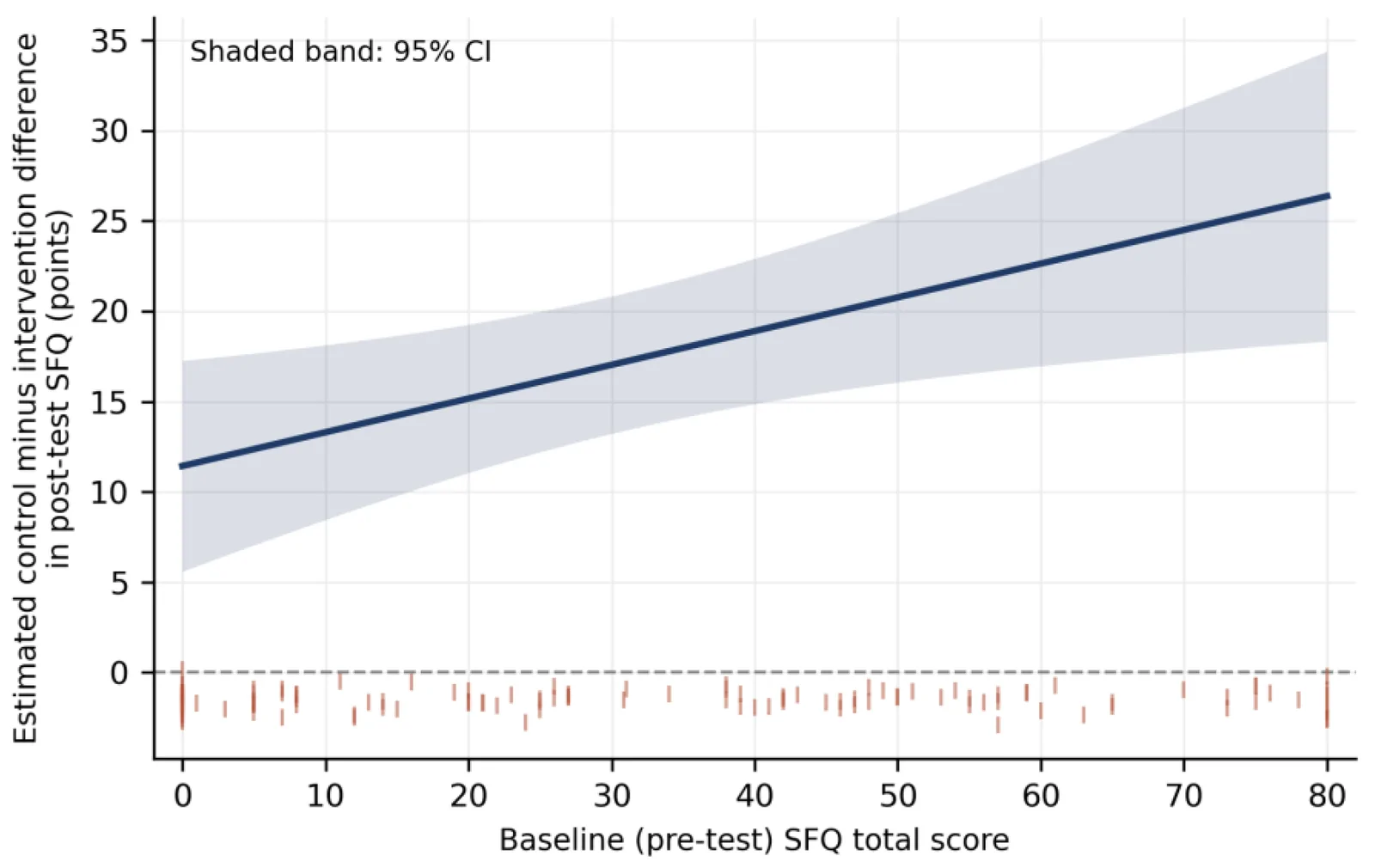
Estimated control minus intervention difference in post-test Surgical Fear Questionnaire score as a function of baseline score, from the interaction model. The shaded band shows the 95% confidence interval and the tick marks along the lower axis show the observed distribution of baseline scores.

SFQ, Surgical Fear Questionnaire; SE, standard error; CI, confidence interval. Group coded 0 = intervention, 1 = control; baseline covariate mean-centred at 30.82. A positive value indicates a higher (worse) score under routine care. Johnson–Neyman analysis indicated that the conditional effect was significant across the entire observed baseline range (SFQ 0 to 80). See also Figure 4.

Because the treatment effect varies with baseline, conditional effects are reported rather than a single adjusted difference (Table 3, Figure 4). The estimated control minus intervention difference rose from 11.43 points (95% CI 5.59 to 17.27) at a baseline score of 0 to 17.03 points (95% CI 13.23 to 20.84) at a baseline score of 30 and 26.37 points (95% CI 18.35 to 34.40) at a baseline score of 80. A Johnson–Neyman analysis showed that the effect was significant across the whole observed baseline range from 0 to 80, so the direction and statistical significance of the finding do not depend on baseline fear; only its magnitude does.

For state anxiety, the homogeneity-of-slopes assumption held (interaction *p* = 0.707), and the common-slope analysis of covariance was therefore appropriate (Table 4). Adjusted means were 30.70 in the intervention arm and 55.57 in the control arm, an adjusted difference of −24.87 points (95% CI −27.33 to −22.41; F(1, 117) = 400.84, *p* < 0.001, ηp^2^ = 0.77). Bootstrap resampling with 10 000 replications produced a closely comparable interval (−24.87, 95% CI −27.19 to −22.48).

In supportive analyses, the mixed analysis of variance showed a time-by-group interaction for both outcomes: F(1, 118) = 65.25, *p* < 0.001, ηp^2^ = 0.36 for surgical fear and F(1, 118) = 429.04, *p* < 0.001, ηp^2^ = 0.78 for state anxiety. For surgical fear, the main effect of time was not significant (*p* = 0.825), because the decrease in the intervention arm and the increase in the control arm cancelled one another.

### 3.4. Subscales of Surgical Fear

**Figure 5:**
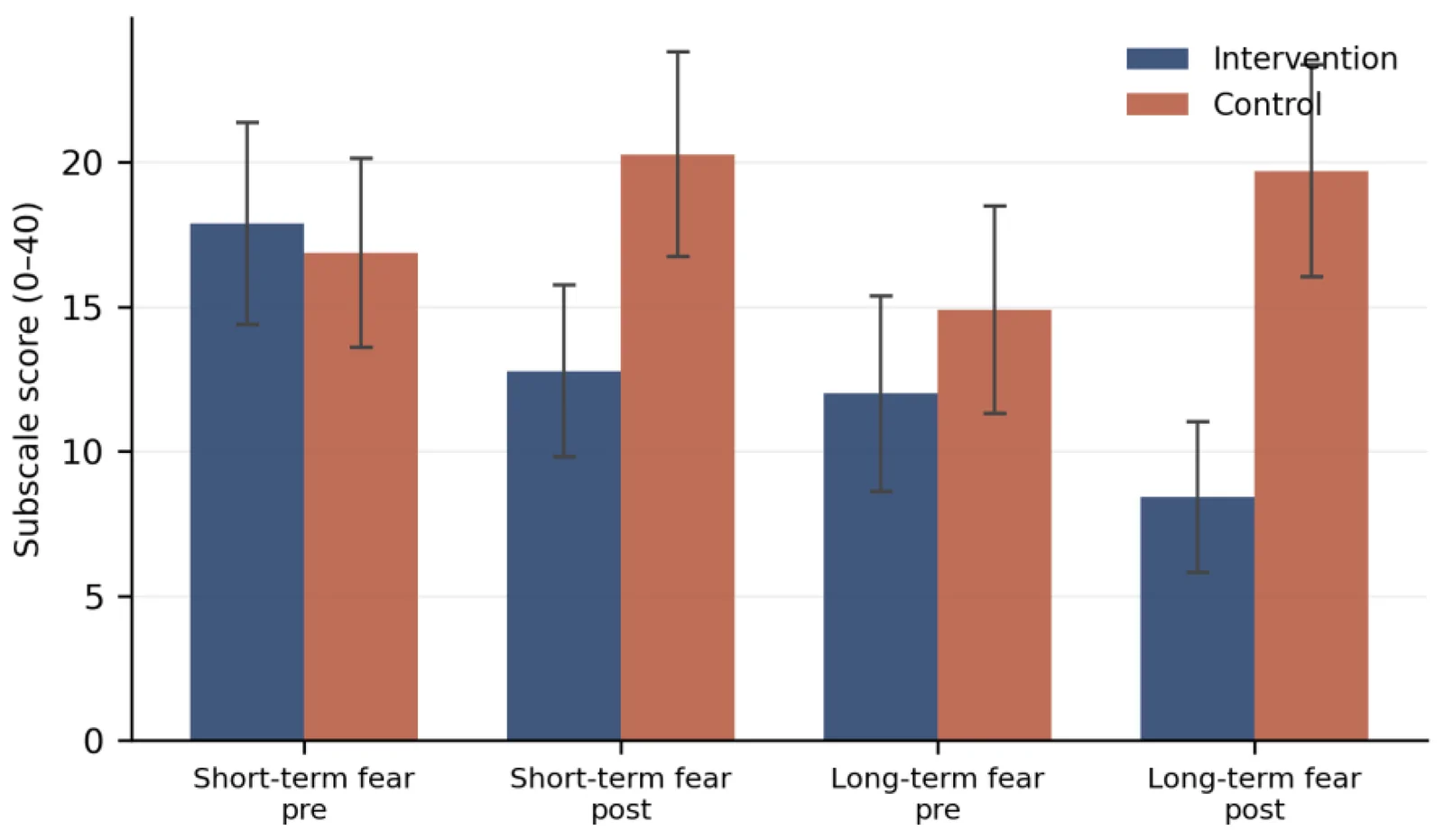
Surgical Fear Questionnaire subscale scores at pre-test and post-test by group. Error bars represent 95% confidence intervals.

The two SFQ subscales behaved consistently with the total score (Table 5, Figure 5). These analyses were exploratory, and the two subscale effects were not formally compared. At pre-test, mean short-term fear was 17.88 ± 13.81 in the intervention arm and 16.86 ± 12.92 in the control arm, and mean long-term fear was 12.00 ± 13.38 and 14.90 ± 14.15, respectively. At post-test, both favoured the intervention arm, with a numerically larger difference for fear of the long-term consequences of surgery (8.42 ± 10.31 vs. 19.70 ± 14.49; g = −0.89) than for fear of its short-term consequences (12.78 ± 11.70 vs. 20.27 ± 13.96; g = −0.58).

### 3.5. Multivariable Analysis

The multivariable model for surgical fear retained the group by baseline interaction, so that the same treatment effect structure identified in Section 3.3 is preserved (Table 6). After adjustment for age, sex, body mass index, and previous surgery, the group coefficient at the mean baseline was 16.76 points (95% CI 12.88 to 20.64, *p* < 0.001) and the interaction remained significant (B = 0.168, 95% CI 0.025 to 0.310, *p* = 0.021); the adjusted R^2^ was 0.857. For state anxiety, where no interaction was present, allocation to the control arm was associated with a post-test score 24.62 points higher (95% CI 21.98 to 27.25, *p* < 0.001). Older age was independently associated with higher post-test scores on both outcomes, whereas sex, body mass index, and previous surgery were not. Variance inflation factors did not exceed 3.19 and Durbin–Watson statistics were close to 2. A further model additionally adjusting for weight-loss method and baseline state anxiety left the group effect essentially unchanged.

### 3.6. Relationship Between Surgical Fear and State Anxiety

**Figure 6:**
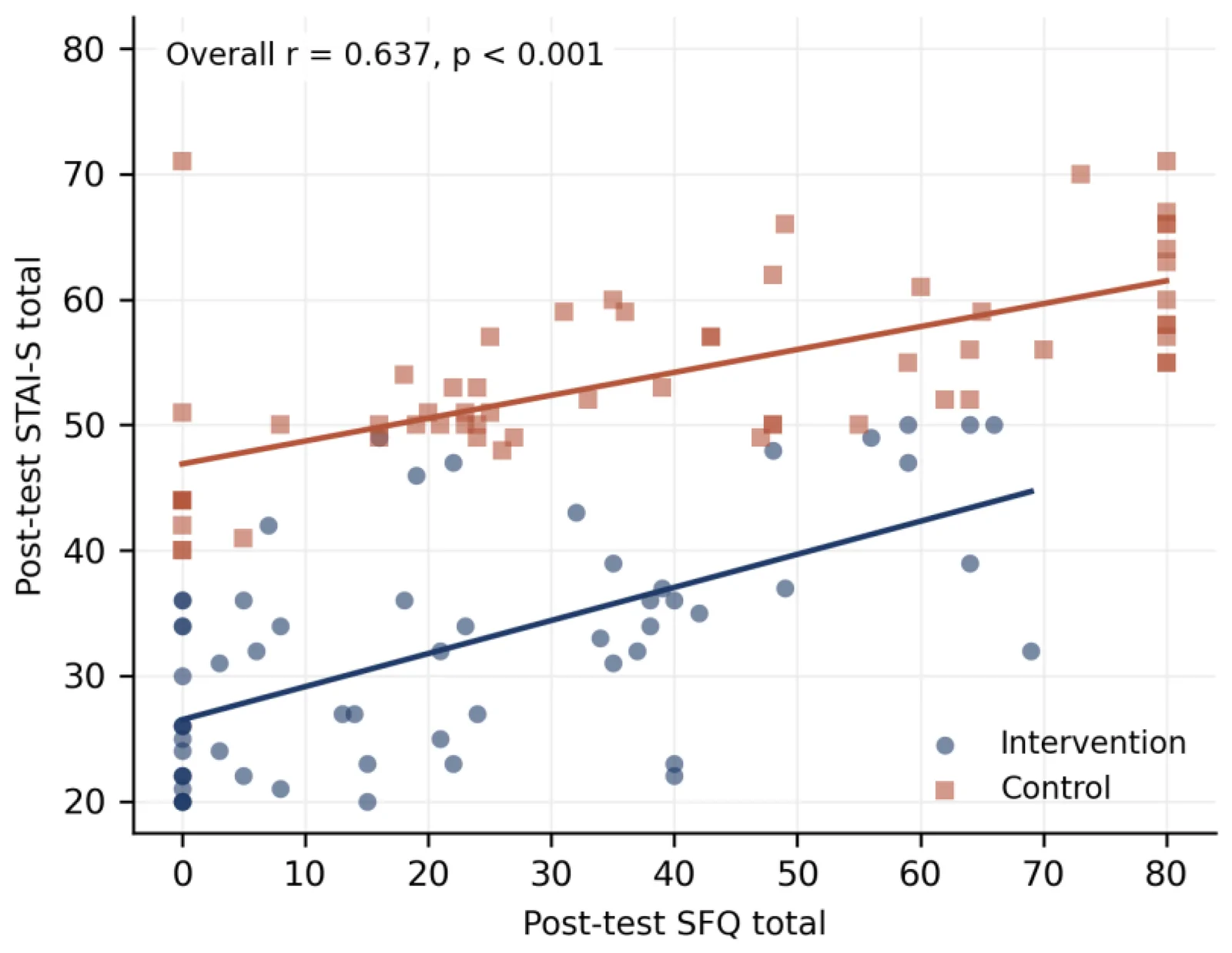
Association between post-test surgical fear and post-test state anxiety, by group, with group-specific regression lines. Circles and the darker regression line represent the intervention arm; squares and the lighter regression line represent the control arm.

Surgical fear and state anxiety were positively and moderately to strongly correlated at both time points and in both arms (Table 7, Figure 6). In the whole sample, the correlation was r = 0.701 (95% CI 0.596 to 0.782) at pre-test and r = 0.637 (95% CI 0.517 to 0.732) at post-test. These analyses were exploratory, and Spearman’s rho gave closely similar values throughout.

Because a cross-sectional correlation between two post-test scores cannot establish that a reduction in fear accompanies a reduction in anxiety within the same individual, the correlation between the two change scores was also examined. In the whole sample, the changes were positively correlated (r = 0.563, 95% CI 0.427 to 0.674), as they were within the control arm (r = 0.508). Within the intervention arm, the two change scores were weakly negatively correlated (r = −0.307, 95% CI −0.520 to −0.058, *p* = 0.017), and this association did not survive a rank-based test (rho = −0.176, *p* = 0.179).

### 3.7. Response Patterns, Individual-Level Change and Sensitivity Analyses

Response patterns in the SFQ were examined post hoc, after inspection of the item-level data showed that many participants had given the same value to every item. At pre-test, 30 of 120 participants (25.0%) responded uniformly, 23 of them scoring zero throughout and 7 giving an identical non-zero value. At post-test, this rose to 54 participants (45.0%), of whom 27 scored zero throughout and 27 gave an identical non-zero value. Fifty-seven participants responded uniformly at one or both time points. The two patterns were not distributed in the same way: uniform zero responding was similar across arms (18 of 60 in the intervention arm vs. 13 of 60 in the control arm, *p* = 0.297), whereas uniform non-zero responding was considerably more frequent in the control arm (20 of 60 vs. 7 of 60, *p* = 0.004). Uniform responding is not in itself evidence of careless or invalid responding, and a score of zero on every item may represent a genuine absence of surgical fear; the analyses below are therefore presented as a test of robustness rather than as a correction for bias.

The primary baseline-adjusted analysis was repeated in the corresponding subsamples so that the same analytical framework applies throughout (Table 8). In the full sample, the adjusted between-group difference was 17.19 points (95% CI 13.29 to 21.08). Excluding all participants who responded uniformly at either time point left 63 patients and gave an adjusted difference of 16.40 points (95% CI 11.25 to 21.56, *p* < 0.001). Excluding only uniform non-zero responders left 93 patients and gave 14.10 points (95% CI 10.07 to 18.13), and excluding only uniform zero responders left 89 patients and gave 19.76 points (95% CI 15.01 to 24.51). The estimated treatment effect on surgical fear is therefore stable across these subsamples once baseline adjustment is applied. The same held for state anxiety, where the adjusted difference in the 63-patient subsample was 22.41 points (95% CI 18.73 to 26.09, *p* < 0.001).

What did not survive was the interaction. The group-by-baseline interaction was absent in every subsample (*p* = 0.845 with all uniform responders excluded, *p* = 0.083 and *p* = 0.241 in the two partial exclusions), indicating that the moderation reported in Section 3.3 depends on participants who responded uniformly and cannot be regarded as an established finding. Internal consistency also fell once uniform responders were removed, from 0.98 to 0.966 at pre-test and from 0.99 to 0.957 at post-test, which suggests that part of the unusually high alpha in the full sample is attributable to this response pattern.

The Reliable Change Index was computed as an exploratory analysis. For state anxiety, the threshold was 6.62 points using Cronbach’s alpha and 11.26 points using the control-arm pre-test to post-test correlation; at these thresholds, 56 of 60 (93.3%) and 49 of 60 (81.7%) intervention patients, respectively, showed reliable improvement, and no control patient improved on either threshold while 30 deteriorated. The classification for state anxiety is therefore robust to the coefficient used. For surgical fear it was not: the threshold was 10.42 points using alpha and 24.28 points using the control-arm correlation, and the number of intervention patients classified as reliably improved fell from 25 of 60 to 4 of 60. Neither coefficient is a satisfactory estimate of test–retest reliability, since the control arm changed significantly over the interval (31.75 to 39.97, *p* < 0.001) and is therefore not a stable reference. The individual-level classification for surgical fear is reported in Supplementary Materials S2 and is not interpreted here.

Several further sensitivity analyses were performed because the SFQ distribution departed from normality (Shapiro–Wilk *p* < 0.001 in both arms) and showed floor effects, with 30.0% of the intervention arm scoring zero at post-test. Non-parametric tests reproduced every significant result. A rank-transformed analysis of covariance gave the same conclusion for both outcomes (*p* < 0.001). Complete-case analysis excluding the single participant with a prorated item, and models additionally adjusting for the baseline-imbalanced variables, left the estimates essentially unchanged.

## 4. Discussion

In this trial, a single 20 min session of immersive 360° virtual nature exposure delivered two hours before laparoscopic sleeve gastrectomy was followed by substantially lower surgical fear and state anxiety than routine care, under which both outcomes increased over the same interval. The difference was independent of baseline scores, of the characteristics that differed between arms by chance, and of age, sex, and body mass index, and it survived every distributional and analytic sensitivity check applied. An important qualification applies throughout what follows. The experimental condition combined immersive audiovisual stimulation, a novel device, 20 min of protected quiet time in a room from which interruptions were excluded, and the undivided attention of a researcher, whereas the control condition contained none of these. The design therefore tests this bundle against routine care and cannot isolate the contribution of the virtual nature content itself. The wording used below refers to the intervention condition rather than to virtual reality as a specific agent.

### 4.1. State Anxiety and the Magnitude of the Observed Effects

The reduction in state anxiety is consistent in direction with the accumulated evidence for preoperative virtual reality. A meta-analysis and meta-regression of virtual reality-enhanced interventions in adults undergoing elective surgery reported a pooled Hedges’ g of −0.76 relative to usual care [14], and randomised trials in elective surgery [20], colorectal and abdominal wall surgery [21], cardiac surgery [23], gynaecological oncology [24] and operating-theatre familiarisation [35,36] have generally reported benefit. The evidence is not unanimous: an earlier meta-analysis found a clear effect in paediatric but not adult populations [19], and effects have been more consistent in children than in adults across the literature more broadly [37,38]. Benefit has nonetheless been documented in several adult populations, including first-time sternotomy [39], dental implant surgery [40] and outpatient gynaecological procedures [41].

The magnitude of the anxiety effect observed here is the central interpretive problem of this trial. The adjusted difference of 24.87 STAI-S points corresponds to g = −2.59 for the post-test contrast, roughly three times the pooled estimate of g ≈ −0.76 from the meta-analysis cited above [14]. A discrepancy of this size is unlikely to reflect a genuinely superior intervention, since the intervention delivered here was simpler than many of those in the pooled trials. Four explanations are more plausible and are not mutually exclusive. First, the comparator: control patients received no substitute activity at all, so the contrast includes the effect of 20 min of individualised nursing attention in a quiet room. In trials using an attention-matched or sham condition, this component is subtracted from the estimate, and pooled estimates are therefore not directly comparable with the present one. Second, demand characteristics: the trial was open-label, both outcomes were self-reported, and the questionnaires were administered by the researcher who had just delivered the intervention, a configuration that maximises the pressure on participants to report improvement. Third, expectancy: patients received a novel technology presented as a means of reducing anxiety, and no attempt was made to control expectancy. Fourth, the mode of outcome administration: the questionnaires were not self-completed but were read aloud item by item by the researcher, who recorded each answer and could see it as it was given, a procedure that requires participants to state their responses aloud to the person who has just delivered the intervention. Taken together, these considerations indicate that the present estimates should be regarded as an upper bound on the effect attributable to immersive nature content, not as a point estimate of it.

Part of the between-group difference is attributable to deterioration in the control arm rather than to improvement in the intervention arm: control patients gained 8.22 SFQ points and 7.15 STAI-S points in approximately 30 min. We previously described this as the natural escalation of anticipatory anxiety as transfer to theatre approaches, but the design does not establish that. The trial has no untreated arm assessed without intervention contact, no third measurement occasion, and no record of what occurred during the interval, so repeated measurement, specific perioperative events in that window, the contrast with an intervention arm receiving visible attention, and genuine temporal escalation cannot be separated. The observation should therefore be reported as a deterioration under routine care in this setting rather than as a demonstrated natural-history effect, and its contribution to the between-group difference should be borne in mind when the magnitude of that difference is interpreted.

### 4.2. Surgical Fear

The effect on surgical fear is the more novel contribution, since most preoperative virtual reality trials have measured general anxiety alone. The findings align in direction with the double-blind randomised trial of virtual reality glasses before open-heart surgery, which reported reductions in surgical fear using the same instrument [22]. The convergence is notable because the intervention content differed, which suggests that reduction in surgical fear may not depend on a single mechanism and that both attentional restoration and procedural familiarisation can move the same outcome.

Two observations refine this picture, and both are exploratory. First, the observed between-group effect size was numerically larger for fear of the long-term consequences of surgery (g = −0.89) than for fear of its short-term consequences (g = −0.58). The two subscale effects were not compared in a formal statistical test, so this descriptive difference is not evidence that the intervention affects long-term fear more strongly. The numerical pattern is nevertheless compatible with an affect-regulation interpretation: by lowering overall arousal, the intervention condition may reduce the catastrophising appraisal that sustains fear about permanent anatomical change, dietary restriction, and body image, concerns that are especially salient in bariatric candidates [6,7]. Second, the estimated difference increased with baseline fear (interaction B = 0.187, *p* = 0.011). This analysis was not the primary study hypothesis, was not the basis of the sample-size calculation, and did not survive exclusion of uniform responders, in whom the interaction disappeared entirely (B = 0.024, *p* = 0.845), although the main treatment effect was essentially unchanged in the same subsample (adjusted difference 16.40 vs. 17.19 points). It may also partly reflect greater scope for change among patients with higher baseline scores, and regression towards the mean. It is reported for completeness and as a hypothesis for prospective testing, and it does not support selective delivery of the intervention in its present form.

### 4.3. Interpretation of the Relationship Between Surgical Fear and State Anxiety

Surgical fear and state anxiety were positively correlated at both time points in both arms (r = 0.61 to 0.79), reproducing the association reported in bariatric [11,12] and other surgical populations [10]. The two constructs are related but not interchangeable, and the present data illustrate why the distinction matters: the intervention condition shifted anxiety almost uniformly but fear more selectively, and within the intervention arm the two change scores were not positively associated. One possible explanation is the restricted variability of the anxiety change scores in that arm, where improvement was nearly uniform; this limited variance reduces the scope for anxiety change to covary with the more heterogeneous change in surgical fear. It is also possible that fear, being anchored to specific and realistic features of the operation, is less amenable to a brief attentional intervention than diffuse state anxiety is. Clinically, this argues against treating a reduction in anxiety as evidence that surgical fear has also been addressed.

### 4.4. Implications for Practice

The intervention tested here is inexpensive, requires no medical prescription, uses a smartphone-based headset costing a small fraction of a dedicated system, and occupies 20 min of nursing time in a window that is otherwise unused. It was well tolerated among those who received it, although tolerability was not universal, since two of the 128 patients assessed reported ocular discomfort when the device was demonstrated and did not take part. What the trial demonstrates, however, is limited to an immediate reduction in self-reported surgical fear and state anxiety measured 10 min after the session, under the conditions tested and against a routine-care comparator. No measurement was obtained at transfer, at induction of anaesthesia, after surgery or during recovery, and no physiological or clinical endpoint was recorded. The associations reported elsewhere between preoperative anxiety and anaesthetic recovery [8] or chronic post-surgical pain [9] motivated the study, but they cannot be used to infer that this intervention improves those outcomes, and no such claim is made. Nor do the present data support screening patients with the SFQ in order to target delivery: the baseline-fear interaction was exploratory, was not robust to the exclusion of uniform responders, and the effect was significant across the entire observed baseline range in any case. The reasonable implication is that a brief structured period of immersive audiovisual distraction and protected quiet time before bariatric surgery merits further evaluation in attention-controlled trials, not that it should be adopted into routine practice on the strength of this trial alone.

### 4.5. Limitations

Several limitations should temper interpretation, and some of them are severe enough to constrain what the trial can support. The configuration of outcome assessment was the least favourable possible in an unblinded trial: the same researcher recruited participants, opened the allocation envelope, delivered the intervention, read each questionnaire item aloud and recorded the responses, and could see each answer as it was given. Interviewer administration requires participants to state their answers aloud to the person who has just delivered the intervention, which is likely to amplify demand characteristics relative to self-completion. In our view, this is the most plausible single contributor to the discrepancy between our effect sizes and pooled estimates from the literature, and it is a principal reason for presenting those estimates as an upper bound. Allocation concealment at the point of enrolment was nevertheless maintained, since the envelope was opened only after eligibility and consent and the allocation list was held by another investigator. The comparator was routine care, not an attention-matched or sham condition, so the specific contribution of immersive nature content cannot be separated from attention, novelty, protected quiet time, and expectancy. The trial was open-label: participants and the interventionist were aware of allocation, and both outcomes were self-reported by patients to the researcher who had delivered the intervention, which risks demand characteristics. Both outcomes were self-reported, and no physiological index of arousal was recorded; heart rate, blood pressure, and other autonomic measures were not collected, so the findings rest entirely on questionnaire responses. This is a meaningful constraint in an unblinded trial, because self-report is the channel through which expectancy and demand characteristics operate most readily, and an accompanying objective measure would have allowed the psychological findings to be corroborated independently.

The trial was registered only after data collection had been completed, so no analysis can be shown to have been prospectively specified beyond the primary outcome named in the ethics application, and the primary and exploratory analyses are distinguished in Section 2.10 on that basis rather than on the basis of a registered protocol. The assumed effect size used in the sample-size calculation was taken from a trial of a different outcome measured after rather than before surgery, as described in Section 2.3.

Internal consistency coefficients for the SFQ were very high (α = 0.98–0.99), higher than in the original validation, and 45% of participants gave identical responses to all eight items at post-test. As shown in Section 3.7, the baseline-adjusted treatment effect was stable when these participants were excluded, but the baseline interaction was not, and internal consistency fell appreciably. Uniform responding is not by itself evidence of invalid responding, and uniform zero scores may reflect a genuine absence of fear; we therefore do not claim that the treatment effect was inflated by it. What the pattern does indicate is that the SFQ discriminated less finely in this sample than its published psychometric properties would suggest, that the moderation analysis rests on those participants, and that the measurement properties of the instrument in bariatric populations deserve further study.

The sample was drawn from a single centre in eastern Türkiye, was predominantly female (71.7%) and almost entirely class II or class III obesity (90.0% class III), and all patients underwent the same procedure. Procedural homogeneity strengthens internal comparability but limits extrapolation to gastric bypass and other bariatric operations, in which the anticipated long-term consequences, and therefore the content of surgical fear, may differ. Patients with a documented psychiatric diagnosis were excluded on the basis of medical record review alone, which both restricts generalisability to a psychiatrically unselected bariatric population and may have imperfectly identified psychiatric comorbidity. Adverse effects were ascertained passively rather than through active elicitation, so the absence of reported effects among participants is weak evidence of safety, particularly as two patients screened before enrolment did report ocular discomfort. Finally, outcomes were measured only 10 min after the session, so it is unknown whether any effect persists to induction of anaesthesia, let alone into the postoperative period.

### 4.6. Recommendations for Future Research

Five priorities follow. First, trials in this population should use an active, attention-matched comparator, such as a non-immersive nature video of equal duration or sham exposure through an inactive headset, to isolate the specific effect of immersion from attention and novelty. Second, blinded outcome assessment should be adopted, with questionnaires administered by an assessor who did not deliver the intervention, together with prospective registration and a published protocol distinguishing confirmatory from exploratory analyses in advance. Third, the psychological outcomes should be paired with objective physiological indices recorded at the same time points; heart rate, heart rate variability, blood pressure, and salivary cortisol are all feasible in the preoperative window and would show whether reported reductions in fear are accompanied by measurable reductions in autonomic arousal. Because these measures are not susceptible to demand characteristics in the way that self-report is, their inclusion would substantially strengthen causal interpretation in a design that cannot be blinded. Fourth, follow-up should extend beyond the immediate post-session window to include measurement at induction and postoperative endpoints such as pain intensity, analgesic consumption, sleep quality, mobilisation, length of stay and satisfaction. Fifth, the baseline-fear interaction observed here should be tested prospectively in a trial powered for it, alongside a dose-finding component comparing session lengths and an examination of whether content type differentially affects the short-term and long-term components of surgical fear. Attention should also be paid to the measurement properties of the SFQ in this population, given the response-set pattern observed, and a cybersickness measure [42] should be included with active adverse-effect elicitation.

## 5. Conclusions

A single 20 min session of immersive 360° virtual nature exposure delivered two hours before laparoscopic sleeve gastrectomy was followed by substantially lower surgical fear and state anxiety than routine care, under which both increased over the same period. The estimated difference in surgical fear was significant across the whole observed range of baseline scores. These effects are much larger than pooled estimates from previous trials, and the design does not permit them to be attributed to the virtual nature content itself: the comparator was routine care rather than an attention-matched condition, the trial was unblinded with self-reported outcomes collected by the person who delivered the intervention, and a marked response-set pattern in the surgical fear data reduced the estimated effect by almost half when it was accounted for. What the trial supports is that a brief, low-cost, well-tolerated, and nurse-deliverable period of immersive audiovisual engagement before bariatric surgery is followed by an immediate reduction in self-reported fear and anxiety. Establishing how much of that reduction is specific to immersive content, and whether it translates into any clinical benefit, requires blinded, registered trials with attention-matched controls, objective physiological measures, and clinical endpoints.

## Figures and Tables

**Table 1:** Baseline sociodemographic and clinical characteristics of the randomised groups (n = 120).

Characteristic	Intervention (n = 60) n (%)	Control (n = 60) n (%)
**Age group**		
20–30 years	24 (40.0)	23 (38.3)
31–40 years	15 (25.0)	19 (31.7)
41–50 years	15 (25.0)	12 (20.0)
51–60 years	6 (10.0)	6 (10.0)
**Sex**		
Female	39 (65.0)	47 (78.3)
Male	21 (35.0)	13 (21.7)
**Marital status**		
Married	41 (68.3)	40 (66.7)
Single	19 (31.7)	20 (33.3)
**Educational level**		
Literate only	4 (6.7)	2 (3.3)
Primary school	11 (18.3)	12 (20.0)
High school	25 (41.7)	30 (50.0)
University	20 (33.3)	16 (26.7)
**Perceived income**		
Income < expenses	7 (11.7)	8 (13.3)
Income = expenses	43 (71.7)	48 (80.0)
Income > expenses	10 (16.7)	4 (6.7)
**Comorbid disease**		
Yes	22 (36.7)	25 (41.7)
No	38 (63.3)	35 (58.3)
**Duration of overweight**		
1–5 years	5 (8.3)	3 (5.0)
6–10 years	26 (43.3)	24 (40.0)
≥11 years	29 (48.3)	33 (55.0)
**Duration of weight-loss attempts**		
1–5 years	21 (35.0)	27 (45.0)
6–10 years	27 (45.0)	21 (35.0)
≥11 years	12 (20.0)	12 (20.0)
**Weight-loss method used**		
Diet only	16 (26.7)	23 (38.3)
Exercise only	16 (26.7)	4 (6.7)
Diet and exercise	28 (46.7)	30 (50.0)
Other or none	0 (0.0)	3 (5.0)
**Body mass index category**		
Obese (class II)	4 (6.7)	8 (13.3)
Morbidly obese (class III)	56 (93.3)	52 (86.7)
**Previous surgery**		
Yes	23 (38.3)	9 (15.0)
No	37 (61.7)	51 (85.0)
**Prior knowledge of VR**		
Yes	34 (56.7)	38 (63.3)
No	26 (43.3)	22 (36.7)
**Prior use of VR**		
Yes	12 (20.0)	11 (18.3)
No	48 (80.0)	49 (81.7)
**Source of surgical information**		
Physician	42 (70.0)	41 (68.3)
Nurse	1 (1.7)	0 (0.0)
Own research	9 (15.0)	10 (16.7)
Peers who had surgery	8 (13.3)	9 (15.0)

VR, virtual reality. Percentages are column percentages. Baseline characteristics are presented descriptively; no significance tests were applied.

**Table 2:** Surgical Fear Questionnaire and State Anxiety Inventory scores at pre-test and post-test, and change from pre-test to post-test, by group (n = 120).

Outcome/Time Point	Intervention (n = 60) Mean ± SD	Control (n = 60) Mean ± SD	t	*p*	Hedges’ g (95% CI)
**SFQ total (0–80)**					
Pre-test	29.88 ± 26.68	31.75 ± 26.70	**n/a**	**n/a**	−0.07 (−0.43 to 0.29)
Post-test	21.20 ± 21.44	39.97 ± 28.20	−4.104 ^b^	**<0.001**	−0.74 (−1.11 to −0.37)
*Change (post − pre)*	−8.68 ± 9.85	8.22 ± 12.87	−8.078 ^b^	**<0.001**	−1.47 (−1.88 to −1.06)
Within-group *p*	<0.001 (dz = −0.88)	<0.001 (dz = 0.64)			
**STAI-S total (20–80)**					
Pre-test	50.83 ± 6.98	47.03 ± 7.68	**n/a**	**n/a**	0.51 (0.15 to 0.88)
Post-test	32.08 ± 9.27	54.18 ± 7.58	−14.300	**<0.001**	−2.59 (−3.08 to −2.11)
*Change (post − pre)*	−18.75 ± 7.75	7.15 ± 5.80	−20.713 ^b^	**<0.001**	−3.76 (−4.40 to −3.12)
Within-group *p*	<0.001 (dz = −2.42)	<0.001 (dz = 1.23)			

SFQ, Surgical Fear Questionnaire; STAI-S, State Anxiety Inventory; SD, standard deviation; CI, confidence interval; dz, Cohen’s dz for paired data. Between-group comparisons: independent-samples *t* test. Within-group comparisons: paired-samples *t* test, reported for completeness only. ^b^ Welch correction applied because Levene’s test indicated unequal variances (*p* < 0.05). All comparisons were confirmed by the Mann–Whitney U and Wilcoxon signed-rank tests (all *p* < 0.001). Bold *p*-values indicate statistical significance at *p* < 0.05; bold row labels are subheadings within the table.

**Table 4:** Baseline-adjusted analysis of post-test state anxiety and time by group interactions.

Analysis	Estimate	95% CI	F	*p*	ηp^2^
ANCOVA, post-test STAI-S (common slope)					
Adjusted difference (intervention minus control)	−24.87	−27.33 to −22.41	400.84	<0.001	0.77
Adjusted mean, intervention arm	30.70				
Adjusted mean, control arm	55.57				
Mixed ANOVA, time by group interaction					
SFQ total			65.25	<0.001	0.36
STAI-S total			429.04	<0.001	0.78

STAI-S, State Anxiety Inventory; SFQ, Surgical Fear Questionnaire; CI, confidence interval; ηp^2^, partial eta squared. For the STAI-S, the homogeneity-of-slopes assumption held (group by covariate interaction *p* = 0.707), so the common-slope model is appropriate; degrees of freedom: 1, 117. A negative adjusted difference indicates a lower (better) score in the intervention arm. Mixed ANOVA degrees of freedom: 1, 118. These analyses are supportive rather than primary.

**Table 5:** Surgical Fear Questionnaire subscale scores at pre-test and post-test by group (n = 120).

Subscale/Time Point	Intervention Mean ± SD	Control Mean ± SD	t	*p*	Hedges’ g (95% CI)
**Short-term fear (0–40)**					
Pre-test	17.88 ± 13.81	16.86 ± 12.92	**n/a**	**n/a**	0.08 (−0.28 to 0.43)
Post-test	12.78 ± 11.70	20.27 ± 13.96	−3.181	**0.002**	−0.58 (−0.94 to −0.21)
*Within-group change*	−5.10 (*p* < 0.001)	+3.41 (*p* < 0.001)			
**Long-term fear (0–40)**					
Pre-test	12.00 ± 13.38	14.90 ± 14.15	**n/a**	**n/a**	−0.21 (−0.57 to 0.15)
Post-test	8.42 ± 10.31	19.70 ± 14.49	−4.915 ^b^	**<0.001**	−0.89 (−1.27 to −0.52)
*Within-group change*	−3.58 (*p* < 0.001)	+4.80 (*p* < 0.001)			

SD, standard deviation; CI, confidence interval. Exploratory analyses. ^b^ Welch correction applied because Levene’s test indicated unequal variances (*p* = 0.003). Bold *p*-values indicate statistical significance at *p* < 0.05; bold row labels are subheadings within the table.

**Table 6:** Multivariable linear regression models predicting post-test surgical fear (interaction retained) and post-test state anxiety (n = 120).

Predictor	SFQ: B (95% CI)	*p*	STAI-S: B (95% CI)	*p*
Control group (ref. intervention)	16.76 (12.88 to 20.64)	<0.001	24.62 (21.98 to 27.25)	<0.001
Baseline score (mean-centred)	0.663 (0.524 to 0.803)	<0.001	0.55 (0.32 to 0.77)	<0.001
Group × baseline score	0.168 (0.025 to 0.310)	0.021	not included	n/a
Age (years)	0.476 (0.150 to 0.802)	0.005	0.23 (0.06 to 0.40)	0.009
Female sex (ref. male)	−0.90 (−5.77 to 3.96)	0.714	−1.50 (−4.30 to 1.29)	0.289
Body mass index (kg/m^2^)	−0.34 (−0.80 to 0.12)	0.146	−0.11 (−0.40 to 0.18)	0.449
Previous surgery (ref. none)	−2.89 (−7.47 to 1.69)	0.214	1.16 (−1.70 to 4.02)	0.424
Model	adj. R^2^ = 0.857; F(7, 112) = 102.75		adj. R^2^ = 0.792; F(6, 113) = 76.47	

B, unstandardised regression coefficient; CI, confidence interval. A positive coefficient indicates a higher (worse) post-test score. The interaction term is retained in the SFQ model so that its structure matches the primary analysis in Section 3.3; the group coefficient is therefore the estimated difference at the mean baseline score. No interaction was present for the STAI-S.

**Table 7:** Correlations between surgical fear and state anxiety, by group and time point.

Group	Pre-Test SFQ—Pre-Test STAI-S r (95% CI)	Post-Test SFQ—Post-Test STAI-S r (95% CI)	Change in SFQ—Change in STAI-S r (95% CI)
Intervention (n = 60)	0.677 (0.511 to 0.794) ***	0.610 (0.422 to 0.748) ***	−0.307 (−0.520 to −0.058) *
Control (n = 60)	0.787 (0.666 to 0.867) ***	0.679 (0.514 to 0.796) ***	0.508 (0.292 to 0.675) ***
Total sample (n = 120)	0.701 (0.596 to 0.782) ***	0.637 (0.517 to 0.732) ***	0.563 (0.427 to 0.674) ***

SFQ, Surgical Fear Questionnaire; STAI-S, State Anxiety Inventory; CI, confidence interval. Pearson correlation coefficients. Exploratory analyses. * *p* < 0.05; *** *p* < 0.001.

**Table 8:** Primary baseline-adjusted analysis of post-test surgical fear repeated in subsamples defined by uniform responding (post hoc sensitivity analysis).

Subsample	n	Adjusted Difference	95% CI	*p*	Group × Baseline *p*
Full sample	120	17.19	13.29 to 21.08	<0.001	0.011
All uniform responders excluded	63	16.40	11.25 to 21.56	<0.001	0.845
Uniform non-zero responders excluded	93	14.10	10.07 to 18.13	<0.001	0.083
Uniform zero responders excluded	89	19.76	15.01 to 24.51	<0.001	0.241

CI, confidence interval. Adjusted difference is control minus intervention, from analysis of covariance of post-test SFQ adjusted for baseline SFQ; a positive value indicates a higher (worse) score under routine care. Uniform responding denotes an identical response to all eight SFQ items at pre-test, post-test, or both. These analyses were post hoc and prompted by inspection of the item-level data.

## Data Availability

The data presented in this study are available on reasonable request from the corresponding author. The data are not publicly available because they contain information that could compromise the privacy of research participants.

## Supplementary Materials

The following supporting information can be downloaded at https://www.mdpi.com/article/10.3390/healthcare14183067/s1, Table S1: Reliable Change Index classification for the Surgical Fear Questionnaire and the State Anxiety Inventory under two alternative reliability coefficients.
