# Supplementary material for: Effect of a Single Immersive Virtual Nature Session on Preoperative Surgical Fear and State Anxiety in Patients Awaiting a Laparoscopic Sleeve Gastrectomy: A Randomised Controlled Trial
Source: Healthcare (Basel). 2026 Sep 17;14(18):3067. doi: 10.3390/healthcare14183067 (PMC13606274; doi:10.3390/healthcare14183067)
Supplement: Supplementary file 1 [file healthcare-14-03067-s001.zip › healthcare-4531815-supplementary.pdf]

Supplementary Materials

Table S1. Reliable Change Index classification for the Surgical Fear Questionnaire and the State Anxiety Inventory under two alternative reliability coefficients

Manuscript: "Effect of a Single Immersive Virtual Nature Session on Preoperative Surgical Fear and State Anxiety in Patients Awaiting Laparoscopic Sleeve Gastrectomy: A Randomised Controlled Trial". These analyses are exploratory and are reported here rather than in the main text for the reasons given below.

Rationale

The Reliable Change Index (RCI) expresses individual change relative to the standard error of measurement, computed as SEM = SD_pre × √(1 − r) and RCI = 1.96 × √2 × SEM, where r is a reliability coefficient. The index is intended to be computed with a coefficient reflecting the stability of repeated measurement. Cronbach's alpha quantifies internal consistency rather than temporal stability, and using it understates measurement error and makes the threshold for reliable change permissive. No independently established test-retest coefficient for the Turkish Surgical Fear Questionnaire in a comparable population was available to us. As an alternative we computed the pre-test to post-test correlation within the control arm, but this is also unsatisfactory, because the control arm did not remain stable over the interval: surgical fear rose from 31.75 to 39.97 ($p < 0.001$) and state anxiety from 47.03 to 54.18 ($p < 0.001$). Neither coefficient is therefore a clean estimate of test-retest reliability. Both are presented so that readers can see how sensitive the classification is to this choice.

**Instrument and reliability coefficient**	**r**	**SEM**	**RCI**	**Intervention arm (n = 60), n (%)**			**Control arm (n = 60), n (%)**		
				**Improved**	**No change**	**Deteriorated**	**Improved**	**No change**	**Deteriorated**
Surgical Fear Questionnaire									
Cronbach's alpha	0.981	3.66	10.14	25 (41.7)	35 (58.3)	0 (0.0)	2 (3.3)	38 (63.3)	20 (33.3)
Control-arm pre-post correlation	0.892	8.76	24.28	4 (6.7)	56 (93.3)	0 (0.0)	0 (0.0)	54 (90.0)	6 (10.0)
State Anxiety Inventory									
Cronbach's alpha	0.902	2.36	6.55	56 (93.3)	4 (6.7)	0 (0.0)	0 (0.0)	30 (50.0)	30 (50.0)
Control-arm pre-post correlation	0.711	4.06	11.26	49 (81.7)	11 (18.3)	0 (0.0)	0 (0.0)	43 (71.7)	17 (28.3)

SEM, standard error of measurement; RCI, Reliable Change Index. SD of the pre-test score was 26.59 for the Surgical Fear Questionnaire and 7.55 for the State Anxiety Inventory.

"Improved" denotes a decrease exceeding the RCI threshold, "deteriorated" an increase exceeding it, and "no change" a change within the threshold in either direction.

For surgical fear the classification is highly sensitive to the coefficient used: the proportion of intervention patients classified as reliably improved falls from 41.7% to 6.7%. For state anxiety it is comparatively robust (93.3% to 81.7%), and no control patient improved reliably on either threshold.

Because neither coefficient is a satisfactory estimate of test-retest reliability, the surgical fear classification should not be interpreted as evidence of reliable individual change and is reported here for transparency only.
